# Enhanced Graphene Based Electronically Tunable Phase Shifter

**DOI:** 10.3390/mi14101877

**Published:** 2023-09-29

**Authors:** Muhammad Yasir, Fabio Peinetti, Patrizia Savi

**Affiliations:** 1 Division of Microrobotics and Control Engineering, Department of Computing Science, University of Oldenburg, 26129 Oldenburg, Germany; 2 Department of Electronics and Telecommunications, Politecnico di Torino, 10129 Torino, Italy; fabio.peinetti@polito.it (F.P.); patrizia.savi@polito.it (P.S.)

**Keywords:** graphene, thick films, tunable devices, phase shifter

## Abstract

In this work, an enhanced tunable microwave phase shifter is presented. The phase shifter consists of three short circuited stubs and a tapered line. The stubs are connected to graphene pads. Graphene’s tunable conductivity is varied by a DC voltage. This in turn causes a reactance variation at the input of the tapered line, which causes a phase variation. The physical parameters of the stubs are optimized for a maximum reactance variation by the help of analytical models, circuit and full wave simulations. Measurements of an optimized prototype are performed and a dynamic phase variation of $59^{\circ }$ is obtained with an amplitude variation of less than 1 dB.

## 1. Introduction

Carbon-based materials have caught significant attention in recent years. This is due to their exciting mechanical, electrical, optical and morphological characteristics [1,2]. Graphene is one of the most researched materials among the carbon-based materials [3]. Graphene is a planar layer of carbon atoms attached to each other in a honeycomb crystal lattice. Some of the most notable properties of graphene are its high mechanical strength, high thermal conductivity and high carrier mobility [4]. It is due to these properties that graphene has been studied in fields ranging from electronics and photonics to mechanics and building and infrastructure [5,6,7,8,9,10,11,12]. There are many applications of graphene in the terahertz band as a tunable adsorber (see, e.g., [13,14]), reconfigurable antennas (see, e.g., [15,16]). In microwave engineering, graphene has been used as a conductive material in the design of antennas [17]. The functionality of graphene as a conductive material in microwave components is limited due to its limited conductivity as compared to conventional materials like copper and gold. Most recently, graphene has been used in the design of tunable microwave components [18]. This has been very useful since the conductivity of graphene can be tuned by the help of a DC voltage. The real part of the conductivity of graphene when varied by the help of an applied DC voltage varies constantly throughout the microwave frequency spectrum up to almost 300 GHz [19]. The imaginary part of the conductivity remains unchanged with the application of a DC voltage. This makes it very easy to deploy microwave passive components like attenuators, antennas and phase shifters [20]. In the design of attenuators the conductivity of graphene placed in different ways on a transmission line is varied by an applied DC voltage in order to achieve tunable insertion loss [21,22,23]. In the case of tunable antennas and phase shifters, the variable conductivity (or resistance when inverted) needs to be converted with the help of microwave circuitry in order to cause a variation of reactance [24,25,26]. This reactance variation can then be used to obtain a phase shift in phase shifters [25,26] or to obtain frequency reconfiguration in tunable antennas [24]. The reactance variation is realized by adjusting the length and width of a stub connected to graphene for increased reactance variation and reduced resistance variation across it when graphene’s conductivity is varied. In this way, a tunable reactance microstrip stub is realized. When placed next to the radiating edge of a patch antenna, it results in a frequency reconfigurable antenna [24], and when placed in the center of a transmission line, it results in a tunable phase shifter [25]. Based on this concept, a number of phase shifters were designed, starting with a graphene phase shifter with one short circuited stub [25] in which the phase shifting capability of the phase shifter was limited and the insertion loss was high. In order to mitigate the insertion loss, a different approach was used with grounded vias in [26]. This approach reduced the insertion loss, but the phase shifting capabilities were still limited. In this paper, an enhanced tunable phase shifter based on graphene is proposed. The phase shifter comprises of three short circuited stubs connected to graphene pads and a tapered line. The paired line is connected to a two port transmission line. The lengths of the stubs are optimized by analytical calculations, circuit models and full wave simulations. The resulting design is fabricated and measured. The phase shifter provides high phase shifting capabilities of $59^{\circ }$ with limited variation of amplitude of 1 dB.

## 2. Materials and Methods

The prototype is designed on the Rogers 3035 RF dielectric substrate (Rogers Corporation, 2225 W. Chandler Blvd., Chandler, AZ 85224, USA). The dielectric substrate has a dielectric constant, $\epsilon_{r}\;=\;3.5$ and a dissipation factor, tanδ = 0.0015 at 10 GHz. The copper thickness is 35 μm and the resistivity $1.68\times 10^{-8}\;\Omega m$. The enhanced tunable phase shifter is fabricated by a photolithographic procedure, which removes excess copper by dissolving it in ferric chloride. Comercial graphene flakes from Nannoinnova, Madrid, Spain, are used to make the deposition. They have a surface area to weight ratio of $45\;m^{2}/g$ with a carbon content of 98.9 wt%. Separately, a solution based on multi-layered graphene nanoplatelets and isopropanol with a concentration of 0.01 g/mL is made. This solution is drop-casted in designated spots on the phase shifter. The isopropanol evaporates at room temperature, leaving behind graphene nanoplatelets suspended in the designated spots. DC and high frequency measurements are simultaneously performed with the help of voltage and current measurement devices, a DC power supply and a two-port vector network analyser (Keysight P9372A Santa Rosa, CA, USA). The DC bias voltage is applied to the phase shifter with the help of a bias tee connected to the SMA connector (RS components, Frankfurt, Germany) of the phase shifter. The bias voltage is applied between the ground plane and the transmission line. The vector network analyzer (VNA) is calibrated with the help of an SOLT calibration with the bias-tees connected in order to remove any impact of the bias-tees on the phase and amplitude of the measurement. After connecting the device to the VNA, different DC voltages are applied and the corresponding scattering parameters are measured. The proposed design of the phase shifter was simulated with the help of the finite-element-modeling-based simulation tool, Ansys HFSS.

## 3. Graphene Flakes Analysis

### 3.1. Raman Analysis

**Figure 1:**
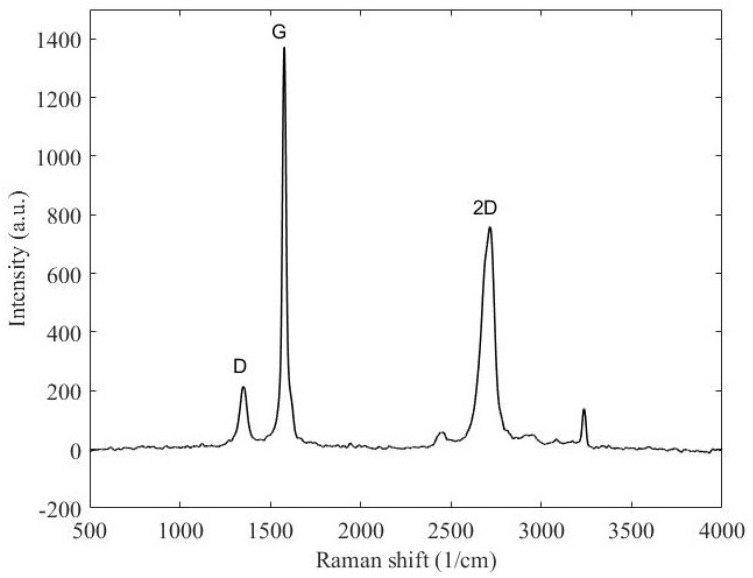
Raman spectroscopy of multilayer graphene sheets.

In the phase shifter fabrication, graphene flakes are deposited as thin films. The Raman spectrum is measured in order to estimate the graphene’s quality [27,28]. The presence of defects and the graphitization grade can be evaluated in a spectral range from $1000\;{\mathrm{cm}}^{-1}$ to $1700\;{\mathrm{cm}}^{-1}$, considering the D and G peaks, respectively, as shown in Figure 1. The second range, from $2200\;{\mathrm{cm}}^{-1}$ to $3500\;{\mathrm{cm}}^{-1}$, contains the second-order Raman spectrum. The G’ (or 2D second-harmonic) peak accounts for overtones of the D vibration mode and the second order product of the D and G peaks. The ratio of fundamental and intermodulation peaks allow for a qualitative estimation of graphene. In particular, Figure 1 shows a much lower D peak than the G peak, meaning the chosen graphene is characterized by a low defect content. Another information that can be inferred from the Raman spectrum is the presence of a multi-layer structure, confirmed by a shoulder on the left of the 2D peak.

### 3.2. Fesem Analysis

**Figure 2:**
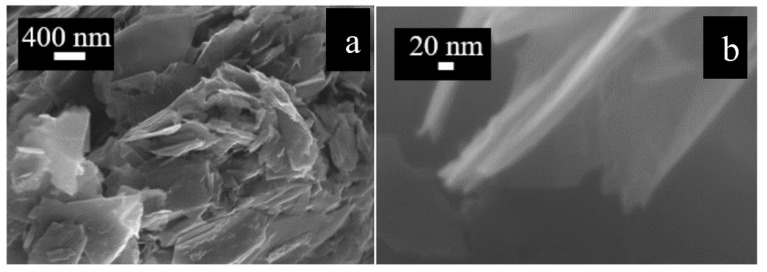
SEM image of graphene flakes with different magnifications: (**a**) magnification of 400 nm, (**b**) magnification of 20 nm. Taken from [26].

Graphene flakes size and surface morphology was investigated by a field emission scanning electron microscope (FESEM, Carl Zeiss AG, Oberkochen, Germany) [26]. The acquired graphene flakes were in the form of a fine black powder, so all the precaution was taken in order to avoid its dispersion in air during characterization. Figure 2 reports different magnifications of the flakes before the deposition. In particular, Figure 2a depicts a three-dimensional structure, showing some agglomerates made up of several sheets of graphene (400 nm magnification). At these magnifications, it is possible to estimate the presence of agglomerates with about 50 nm in thickness. A further magnification (20 nm) is reported in Figure 2b, where a multi-layered nanostructure can be seen in detail. The average flake size is in the range of 0.5 μm.

## 4. Results

**Figure 3:**
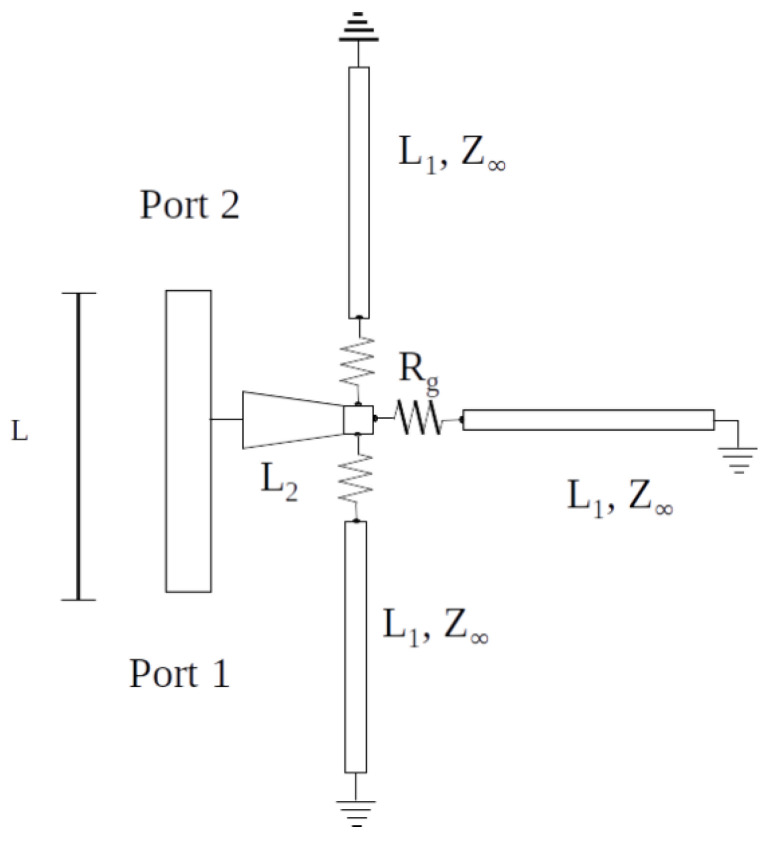
Schematic diagram of the three-stub structure with graphene modeled as resistance.

The phase shifter consists of three short circuited stubs connected to graphene pads and a tapered line. Graphene is modeled as a resistor with resistance equal to $R_{g}$. The tapered line is connected to a two-port transmission line. A schematic diagram of the tapered line and the short-circuited stubs interconnected through graphene is shown in Figure 3. $L_{1}$ is the length of the stubs, supposed to be of the same length. Such a value is optimized in the following discussion. $L_{2}$ is the taper length.

An ideal phase shifter should have a maximum phase variation and a minimum insertion loss variation. To this aim, the variation of the imaginary part of the input impedance $Z_{in}$, needs to be maximized whereas the variation of the real part of $Z_{in}$ needs to be minimized. The input impedance of each of the short-circuited stubs $Z_{1}$. The impedance of the and the three stubs in parallel is given by $Z_{L}$.

(1) $$Z_{L}=\frac{R_{g}+jZ_{1}\tan\left(\beta L_{1}\right)}{3}.$$

The impedance at the input of the tapered line section can be simplified by using a transmission line model. The input impedance at the beginning of the tapered line is given by $Z_{in}$:

(2) $$Z_{in}=\frac{Z_{L}+j\tan\left(\beta L_{2}\right)}{1+jZ_{L}\tan\left(\beta L_{2}\right)}$$

**Figure 4:**
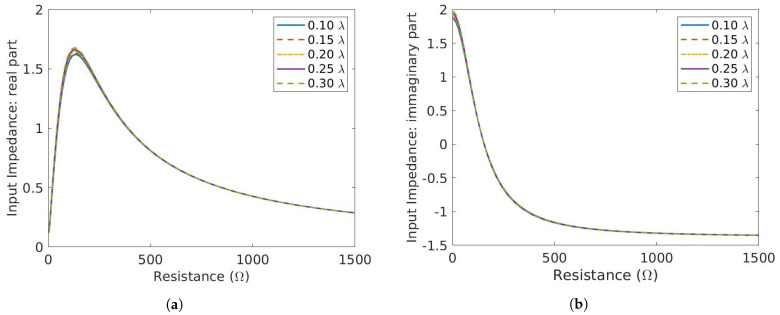
Normalized Input impedance of the three-stub structure computed analytically. (**a**) Real part. (**b**) Imaginary part.

**Figure 5:**
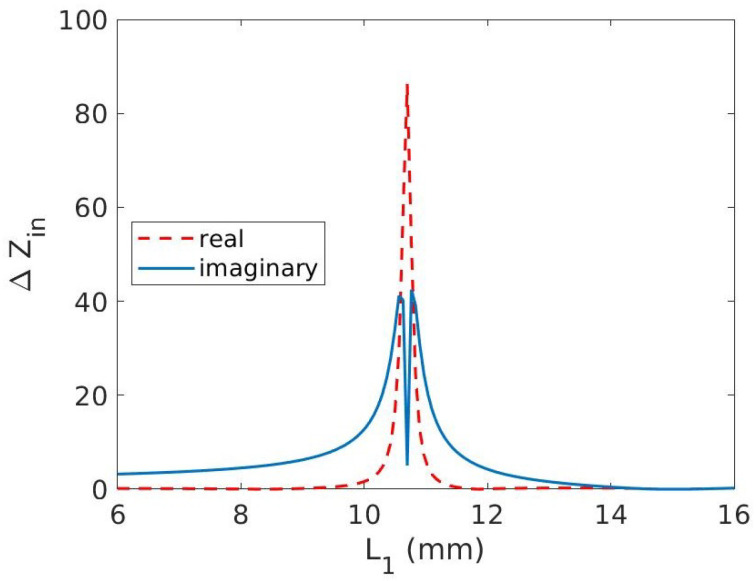
Normalized differential input impedance of the three-stub structure.

**Figure 6:**
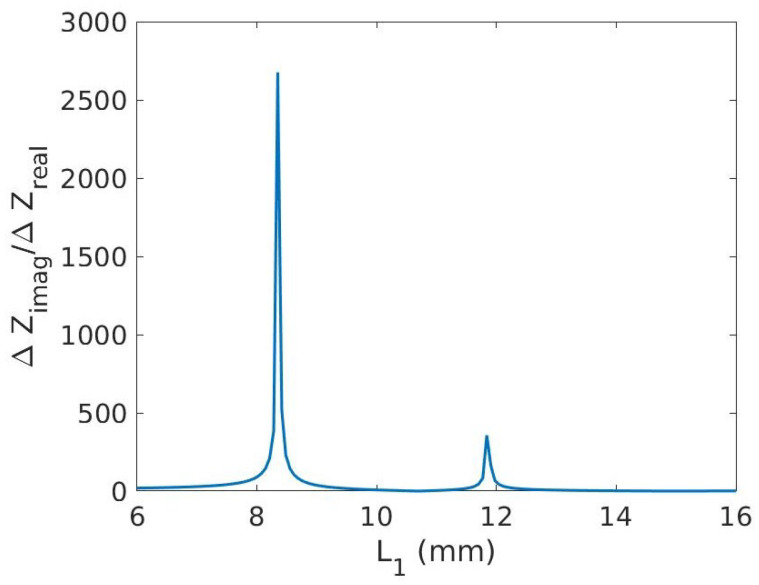
Ratio of the imaginary to real impedance of the three-stub structure.

The lengths of the stubs can be optimized for the range of graphene resistance variation in order to maximize $ℑ\{Z_{in}\}$ and minimize $ℜ\{Z_{in}\}$. Therefore, $ℜ\{Z_{in}\}$ and $ℑ\{Z_{in}\}$ is computed for stub lengths (L) varying from 0.10 λ to 0.30 λ according to Equation (2). A plot of $ℜ\{Z_{in}\}$ and $ℑ\{Z_{in}\}$ is shown in Figure 4a and Figure 4b, respectively. In order to quantify the variation in each of the components of $Z_{in}$, $\Delta Z_{in}\;=\;Z_{in}{{|}_{V_{appl}}-Z_{in}|}_{0V}$ is shown in Figure 5. It can be seen that by increasing the length of the stubs, both $\Delta ℜ\{Z_{in}\}$ and $\Delta ℑ\{Z_{in}\}$ increase albeit with different intensity up to 10.8 mm. $\Delta ℜ\{Z_{in}\}$ peaks at slightly higher $L_{1}$ than $\Delta ℑ\{Z_{in}\}$. In order to analyze and optimize the stub length, it is necessary to identify the length for which the ratio $\Delta ℑ\left\{Z_{in}\right\}/\Delta ℜ\left\{Z_{in}\right\}$ is maximized. This condition ensures that a minimum variation in the real part corresponds to a maximum variation in $ℑ\{Z_{in}\}$. To this aim, the ratio $\Delta ℑ\left\{Z_{in}\right\}/\Delta ℜ\left\{Z_{in}\right\}$ is shown in Figure 6. The ratio shows two peaks at $L_{1}=\;8$ mm and $L_{1}=\;12$ mm, respectively. The peaks are due to low values of $ℑ\{Z_{in}\}$ and even lower values of $ℜ\{Z_{in}\}$. If the lengths of the stubs were chosen at the two peaks, the eventual phase shifter would have very little phase shifting capabilities. Taking a look at Figure 5, two other options to maximize phase shifting can be identified as $L_{1}=10$ mm and $L_{1}=11.5$ mm. The plot in Figure 5 is not symmetrical, resulting in a more convenient choice for $L_{1}=11.5$ mm, where the curves are slightly steeper than for $L_{1}=10$ mm. For $L_{1}=11.5$ mm, the high value of $Re\{Z_{in}\}$ can thus be tolerated if the phase shifting capability has to be maximized. The high value of $Re\{Z_{in}\}$ might result in a slightly higher insertion loss. Please note that this analysis is an approximation of the input impedance of the three stub structure, therefore does not take into account certain losses, for example dielectric losses, conductor losses, etc.

**Figure 7:**
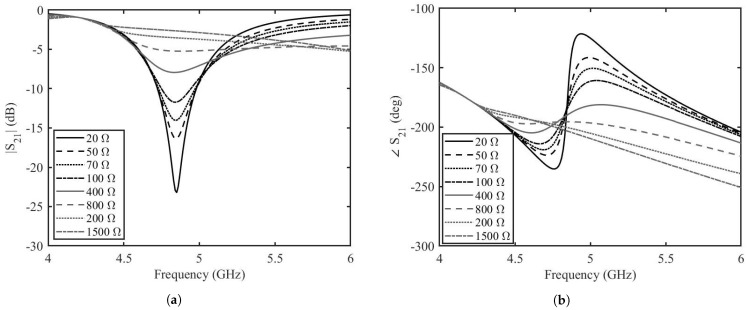
Transmission coefficient of the enhanced graphene phase shifter based on the circuit model simulations. (**a**) amplitude, (**b**) phase.

**Figure 8:**
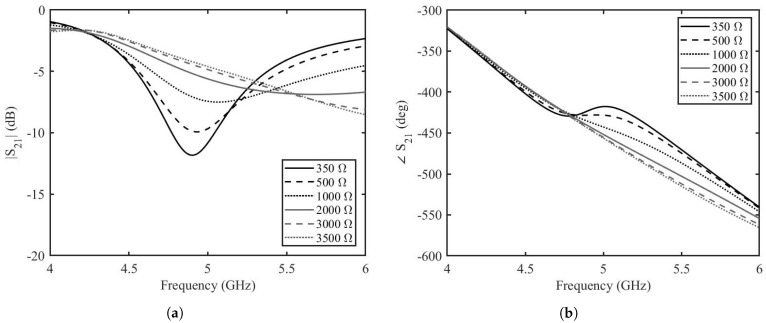
Transmission coefficient of the enhanced graphene phase shifter based on the FEM simulation. (**a**) amplitude, (**b**) phase.

Once the length of the stubs is optimized by this analytical analysis, each stub is connected to a two-port transmission line. The topology is simulated for different values of graphene resistances by the help of AWR Microwave Office (Cadence Design Systems, Inc. 2655 Seely Avenue, San Jose, CA 95134, USA). For the sake of simplicity, the transmission lines are modeled as ideal, lossless transmission lines, the tapered line section is modeled as a non-homogeneous linear taper and graphene is modeled as a tunable resistor. The two-port transmission line has a characteristic impedance of 50 Ω and an electrical length of $180^{\circ }$. The tapered line has a length of 6 mm and widths of 3.3 mm and 1 mm. The stubs have a characteristic impedance of 100 Ω and length of $107^{\circ }$. The amplitude and phase of the transmission scattering parameters resulting from the circuit analysis of depositions with resistances ranging from 20 Ω to 1500 Ω are shown in Figure 7a,b. It can be seen that at the frequency of interest of 5 GHz, the variation in amplitude of the transmission is almost −5 dB, whereas the variation in phase is $80^{\circ }$. This shows that there is great potential in the phase shifting capabilities of the phase shifters, but further analysis needs to be made before prototype fabrication and measurements. The proposed design of the phase shifter was simulated by the help of the finite element modeling based simulation tool, Ansys HFSS. The prototype is designed on the Rogers 3035 RF dielectric substrate. The dielectric substrate has a dielectric constant, $\epsilon_{r}\;=\;3.5$ and a dissipation factor, tanδ = 0.0015. The length of the two-port transmission line is 45 mm. The tapered line has length of 6 mm and widths of 3.3 mm and 1 mm. The stubs have widths equal to 1 mm and lengths of 11.55 mm including the top of the grounded via. The width of graphene deposition is 1 mm and the length is 0.2 mm. The reduced length of the graphene pad helps in reaching lower resistance values since the resistance of graphene depends on the aspect ratio. The finite element modeling (FEM) simulated amplitude and phase for the graphene resistance from 350 Ω to 3500 Ω are shown in Figure 8a,b. The amplitude variation of the transmission scattering is less than in the case of the analytical calculations and in the equivalent circuit simulations. The variation of the phase is not as high as in the circuit simulations, but it is retained over a long frequency band. There is a slight shift in the resonance frequency of the phase shifter, but this is expected as the circuit model is simplistic. The amplitude variation is less than 2 dB at 5.2 GHz with a phase variation of more than $50^{\circ }$.

**Figure 9:**
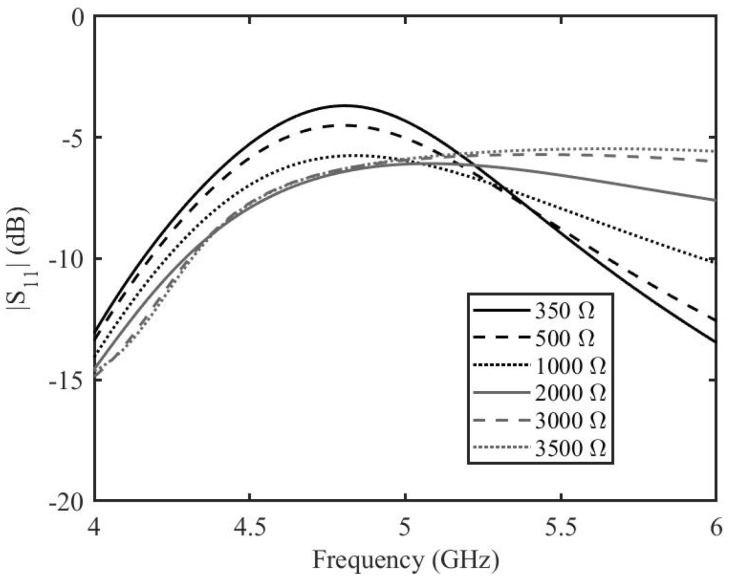
Reflection coefficient amplitude of the enhanced graphene phase shifter based on the FEM simulation.

**Figure 10:**
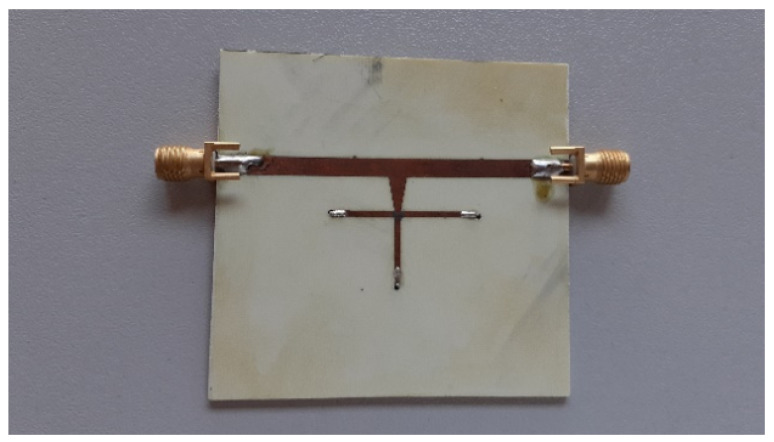
Prototype of the tunable phase shifter.

The reflection coefficient (see Figure 9) is around −5 dB at 5 GHz, which is a bit on the higher side; however, the phase shifter is designed to operate at narrowband and therefore can be used at frequencies above or below 5 GHz. The reflection coefficient values are drastically degraded outside 5 GHz. Please note that the phase shifting capability of the phase shifter covers a comparatively wider band. A fabricated prototype is shown in Figure 10.

**Figure 11:**
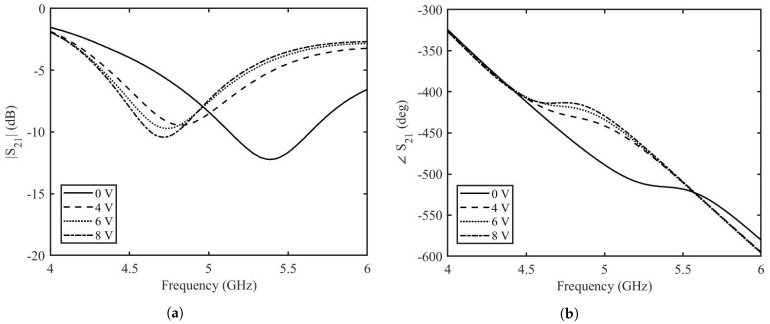
Measurements of transmission coefficient of the enhanced graphene phase shifter; (**a**) amplitude, (**b**) phase.

**Figure 12:**
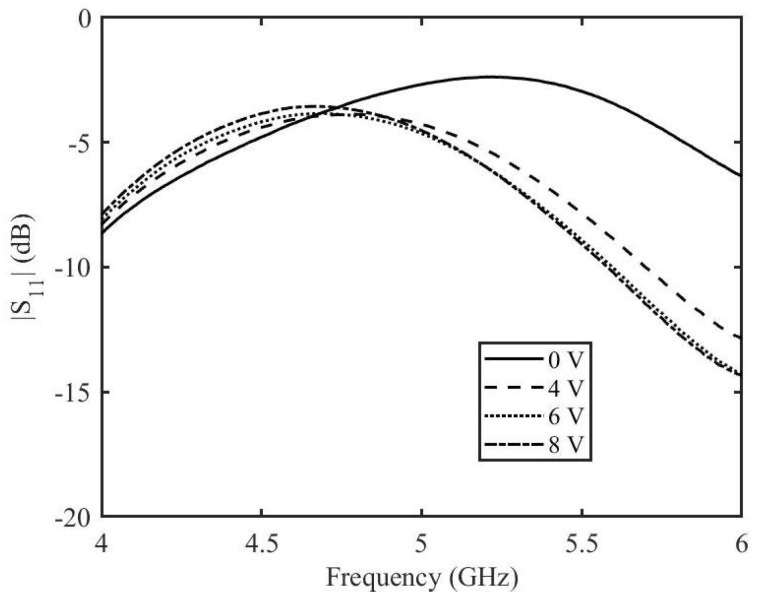
Reflection coefficient amplitude of the enhanced graphene phase shifter based on measurements.

The measured amplitude and phase of the two port scattering parameters at different DC voltages are shown in Figure 11. It can be seen that the variation in amplitude for all the applied DC voltages is minimum whereas there is a significant variation of phase when the applied DC voltage is varied. At the frequency of 5 GHz, at 0 V applied DC voltage, the amplitude is −8.4 dB and the phase is ${-488.6}^{\circ }$. At the maximum applied DC voltage of 8 V, the amplitude is −7.4 dB and the phase is ${-429.2}^{\circ }$. This result in a variation of the amplitude of $S_{21}$ of 1 dB and a phase variation of $59^{\circ }$. The reflection scattering parameters for different applied voltages are shown in Figure 12. The higher values of reflection for some voltages at certain frequencies can be correlated to the insertion loss of the phase shifter. A reduction in the reflection loss can be achieved by choosing a length of the stubs for which the value of $ℜ\{Z_{in}\}$ is smaller. However, this will impact the phase shifting capabilities of the phase shifter.

## 5. Conclusions

An enhanced tunable phase shifter based on graphene is presented. The phase shifter has the capability to dynamically tune its phase by a variable DC voltage. The phase shifter is based on three short circuited stubs connected to a tapered line and a two-port transmission line with the help of graphene pads. In order to maximize the phase shifting capability of the phase shifter and to reduce the insertion loss, the imaginary input impedance at the tapered line section needed to be maximized, keeping the real part as low as possible. Analytical computations are performed in order to choose appropriate lengths and widths of the stub. The values achieved are verified by further analysis, performed with circuit models and finite element simulations. This is followed by the fabrication and measurement of prototypes with the optimized parameters. The optimized design has resulted in enhanced phase-shifting capabilities with minimum insertion loss variation.

## Data Availability

Not applicable.
